# Report of the Distribution Committee

**Published:** 1917-12-22

**Authors:** 


### KING EDWARD'S HOSPITAL FUND—(*continued*).

### REPORT OF THE DISTRIBUTION COMMITTEE.

(1) The Governors and General Council have this year fixed the sum available for distribution amongst the London hospitals at £181,000, as against £162,500 in 1916, and £133,500 in 1915.

(2) The number of hospitals applying for grants is again 107.

(3) Of the total amount distributed, £151,925 has been applied in the form of grants to maintenance. This is an increase of £8,500 over the corresponding total in 1916, and of £34,400 over 1915.

### Helping the Hospitals which Help Themselves.

(4) Now that the war has lasted over three distribution periods its effect on the finances of the hospitals is less difficult to estimate. Although the allocation of the increased maintenance grants is necessarily based each time on audited accounts which are nearly a year old, it has been found that over the whole period the grants have been adjusted fairly closely to the requirements of the various hospitals.

In making these awards, the Distribution Committee have not lost sight of the principle of helping the hospitals which have maintained or improved their position by their own efforts. Last year several exceptional grants were made in reduction of debt, after a careful consideration of the steps already being taken to secure local and general support. This year several additional grants are made to supplement the amounts collected by special efforts, sometimes made at the direct instance of the Fund, and resulting in substantial improvement in the financial position at the close of the year.

### Increase in Capital Grants.

(5) The capital grants this year amount to £29,075, an increase of £10,000 as compared with 1916, or of £13,100 as compared with 1915. Of this year's total, only £4,025 is in respect of schemes sanctioned during the war on the ground of exceptional urgency, while £20,400 is in reduction of capital liabilities incurred before the war. The remaining £4,650 has been earmarked for schemes of improvement which cannot be carried out until after the war, but which are so urgent that, in the opinion of the Committee, it is desirable that plans should be ready and a certain amount of funds available, so that the work may be put in hand without delay as soon as circumstances permit.

### Building Schemes during the War.

(6) At the same time, the Committee have continued to discourage the issue during the war of appeals to the public in aid of building schemes not of this degree of urgency. The request of the General Council that all hospitals contemplating any considerable reconstruction or extension should, before taking definite action, submit their proposals to the Fund, still holds good; and the issue of a public appeal is considered by the Committee to be definite action.

### Charing Cross Hospital Free of Debt.

(7) The Committee recommend with special satisfaction the grant of £5,000 to extinguish the mortgage debt on Charing Cross Hospital. Two years ago this debt stood at £85,000, with a provision of only £7,800 towards its redemption; and the position of the hospital, as the result of premature extension, had been rendered so precarious that the Fund only felt justified in continuing its support "in view of the new management." At the beginning of the year 1914 the debt was still close on £50,000. The Chairman and Committee are to be congratulated on having reduced this debt to £5,000 during the war, while

**KING EDWARD'S HOSPITAL FUND**—(*continued*).

at the same time largely increasing the current income. The grant of £5,000 by the Fund (making £10,700 in all contributed to this object) will thus enable the hospital to begin the new year free of debt.

(8) The position with regard to the amalgamation of the throat, nose, and ear hospitals, so far as the Fund is concerned, is the same as last year. The Committee have again decided to recommend an extra grant to the amalgamated Hospital for Diseases of the Throat to assist in meeting additional expenditure resulting from the postponement of the building scheme in consequence of the war.

(9) The Committee have as usual taken into consideration the returns made by the hospitals relating to the treatment of naval and military patients during the previous year, and also during the first nine months of the current year.

(10) The following grants in aid of schemes of building expenditure which have been deferred during the war are recommended for renewal: Hospital and Home for Sick Children, Sydenham (1915), £75 to central heating; Queen Charlotte's Lying-in Hospital (1913), £1,000 to new out-patient department; St. Mary's Hospital for Women and Children, Plaistow (1913), £1,500 to new nurses' home and out-patient department.

(11) The Committee have borne in mind the importance of economy of management in the working of a hospital in all its departments, and though they recognise that wages, salaries, and the cost of provisions, drugs, coal, etc., have inevitably risen during the war, they have continued to draw attention to cases where the total cost per bed is exceptionally high, or where the rise is considerably greater than at other comparable hospitals.

Special Grants Recommended.

(12) The Visitors' Reports have again proved to be of the greatest service. The Committee frequently direct the special attention of all the visitors in a given year to some particular point. This year the questions selected for more detailed inquiry than usual related to nurses' accommodation. The Committee were glad to see that in the great majority of instances the reports were satisfactory, and wherever improvements seemed *prima facie* desirable and practicable a private remark has been appended to the grant, asking for the observations of the Committee of Management. In more than one case special grants are recommended in aid of urgent reconstruction.

The details of the awards to the various hospitals are appended.

For the Committee, W. S. Church, Chairman.
December 1917.

